# The Evaluation of Meat and Carcass Characteristics of Thin‐ and Fat‐Tailed Lambs Slaughtered at 40 kg According to EUROP Classification System

**DOI:** 10.1002/vms3.70449

**Published:** 2025-06-06

**Authors:** Bulent Teke, Akif Uysal, Mustafa Ugurlu, Buket Nacar, Deniz Ay, Filiz Akdag

**Affiliations:** ^1^ Department of Animal Breeding and Husbandry Ondokuz Mayis University Veterinary Faculty Atakum Turkey

**Keywords:** conformation class, EUROP carcass classification, fatness class, fat‐tailed lamb, meat quality

## Abstract

The study aimed to evaluate the effectiveness of the Europ carcass classification system (ECCS) in discriminating between carcass characteristics and meat quality of fat‐tailed (FT) and thin‐tailed (TT) lambs. In this study, 45 single male lambs of the breeds Akkaraman (*n* = 14), Karayaka (*n* = 15), and Herik (*n* = 16) were used. The lambs were fed and slaughtered at 40 kg. After analysis, two groups were obtained in respect of meat quality and carcass characteristics. One was Akkaraman and Herik as FT, and the other was Karayaka. The effect of fatness class (FC) on carcass characteristics in FT and TT breeds was generally significant. The effect of FC on meat quality characteristics was significant only in a* and b* and expressed juice traits in TT lambs, while no meat quality parameters were affected in FT lambs. While the effect of conformation on carcass traits was significant in terms of trimmed meat, bone and fat percentages in FT lambs, the effect of conformation class (CC) on meat quality traits was insignificant in both tail structures. In conclusion, FC is more effective than CC in distinguishing carcass and meat quality traits in FT and TT lambs according to EECS. This system could be improved especially meat quality characteristics both FT and TT lamb carcasses.

## Graphical abstract

The European carcass classification system was originally developed for European thin‐tailed sheep breeds. Since the distribution of fat tissue in thin‐tailed lambs differs from fat‐tailed lamb, this may create a disadvantage regarding the effectiveness of the EUROP classification system for fat‐tailed breeds.

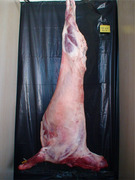

## 1 Introduction

Evaluating carcass characteristics is an important step in establishing quality and value when an animal is slaughtered (Delgado‐Pando et al. 2021). Classifying and grading procedures provide a better understanding of livestock products and market trends. This allows for system refinements that guarantee the quality and homogeneity of the product to ensure competitiveness (Lopez‐Campos et al. 2019). There are various carcass evaluation and grading methods used in the world. The evaluation systems may vary from country to country. Some systems are focused on estimating yield, while others consider quality characteristics. The Europ carcass classification system (ECCS) is utilised to categorise carcasses of various farm animals in the countries of the European Union (Delgado‐Pando et al. 2021).

The ECCS was initially designed to assess the quality of lamb carcasses from thin‐tailed (TT) lambs breeds reared in European countries. The lamb carcasses are classified according to two schemes in the European Union. They depend on whether the carcass weight is over or less than 13 kg. There are three main price criteria for lambs with a carcass weight of 13 kg or more carcass weight: fatness, conformation and weight. The conformation class (CC) is a morphological classification system that describes carcass shape in terms of convex or concave profiles. One of its main functions is to indicate the proportion of meat to bone. In this context, flesh or meat is the sum of fat and lean. Fatness class (FC) is utilised to indicate the level of visible adipose tissue on the surface of the carcass, such as subcutaneous fat. A carcass with well‐developed, rounded muscles is associated with a high conformation score. A high FC identifies a carcass with high subcutaneous fat (Johansen et al. 2006). In Turkey, slaughterhouses do not use carcass grading or grading systems to price lamb carcasses. Pricing varies according to sheep age, carcass weight and carcass visual fat condition. There is an expectation that national current legislation in Turkey on the classification of sheep carcasses will be line with EU legislation (Ekiz et al. 2021).

Turkey's sheep population is 44.7 million heads, and about 90%–95% of the total sheep population are fat‐tailed (FT) sheep (FAOSTAT 2022). FT sheep are characterised by fat accumulating in the tail. Fat deposits in the tail provide important energy, when feed is scarce. The sheep are able to move long distances and cope with harsh weather condition, such as elevated temperatures and high humidity (Kashan et al. 2005; Teke et al. 2017). Turkey has a large number of native sheep breeds which have adapted well to its climate and feed resources. TT breeds of sheep live along the coast, while FT sheep live in the inland of the country. In Turkey, about 45% of the sheep population is of the Akkaraman breed, while 6% are of the Karayaka and Herik breeds. Among these breeds, the Karayaka breed is TT, while the Akkaraman and Herik breeds are FT (Akcapınar 2000).

Several researchers have reported that the ECCS does not distinguish in terms of CC and FC for carcass and meat quality traits, is deficient in the classification of meat quality traits and needs improvement (Stanisz et al. 2012; Janiszewski et al. 2018; Nogalski et al. 2019; Ekiz et al. 2024). Almost all of these studies were conducted on TT breeds and carcasses weighed less than 13 kg (Velasco et al. 2000; Cañeque et al. 2001; Santos‐Silva et al. 2002; Díaz et al. 2003; Tejeda et al. 2008; Miguel et al. 2003; Ciliberti et al. 2021). On the other hand, as the distribution of adipose tissue in TT lambs differs from that of FT lambs, this may be a disadvantage in the effectiveness of ECCS for FT breeds. There is limited information in the literature on whether the ECCS is discriminative in FT lambs, which have a different fat distribution in comparison with TT lambs. The present research aimed to evaluate the effectiveness of the ECCS in discriminating between carcass characteristics and meat quality of FT and TT lambs.

## 2 Material and Method

### 2.1 Animal Management and Treatments

The research was carried out at the Research and Application Farm of Agricultural Faculty of the University of Ondokuz Mayis. In this study, 45 single male lambs born in February 2022 from three breeds were Karayaka (*n* = 15), Akkaraman (*n* = 14) and Herik (*n* = 16), ∼20 kg live weight. Lambs of each breed were randomly selected from ∼100 lambs. Each breed was placed in a random compartment. Stocking density (0.7 m^2^ per lamb) and feeder management (30 cm feeder per lamb) were appropriate during the experimental period. The lambs were vaccinated against clostridial disease and given anthelmintic treatment against parasitic diseases before the fattening period. Before the fattening phase began, the lambs were gradually adapted to the high‐concentrate diets using a 15‐day step‐up protocol. The concentrate and forage were applied as a mixed ration. The concentrate and alfalfa hay were ground and passed through a 1‐mm sieve. Next, dry matter, ash, crude protein and ether extract were analysed to determine their chemical composition according to the AOAC methods (Association of Official Analytical Chemists 1995). Analysis results are provided in Table 1. Lambs had ad libitum access a concentrate and alfalfa hay diet which met nutritional requirements recommended by the National Research Council (NRC) (NRC 2007) until they reached a slaughter weight of 40 kg. Free access to water was also provided. The lambs were not fed but were permitted ad libitum access to water for the 16 h preceding the slaughtering process. Pre‐slaughter weights (PSW) and ultrasonic measurements of the lambs were recorded before slaughtering.

**TABLE 1:** Nutrient contents of the concentrate and roughage used for the lambs during the fattening period.

Nutrient content	Concentrate	Alfalf^a^
Dry matter, %	89.74	90.19
Crude protein, %	16.05	17.25
Ether extraction, %	3.77	2.09
Ash, %	7.20	10.11
Neutral detergent fibre, %	22.93	38.89
Acid detergent fibre, %	12.12	29.23
Metabolic energy, kcal/kg	2844.00	2031.54

Ultrasound measurements were collected using a portable real‐time ultrasound device (Aloka SSD‐500) with a 3.5‐MHz, 12.5‐cm linear transducer. Wool was cut from the measuring areas, and acoustic gel was applied. The transducer was placed 3–4bL, lateral and parallel to the spine, after physical palpation and preparation. The measurements were obtained on the left side and 4 cm from the spinal column. Once the scan had been carried out, the thickness, width and areas of muscle, subcutaneous fat thickness were measured using the scanner's electronic callipers with a resolution of 0.1 cm. Muscle surface area was measured ultrasonically in live animals on the same image after the muscle borders were drawn (Akdag et al. 2015).

All lambs were individually weighed each week during the fattening period and slaughtered when they reached 40 kg live weight. The slaughter age of Akkaraman, Herik and Karayaka was 75.57 ± 5.73, 89.13 ± 7.59 and 101.27 ± 9.47 days, respectively. The lambs were slaughtered without stunning by the halal procedure under routine commercial practice in Turkey. Head, skin, feet, gastrointestinal tract, testicles and offal were removed after the slaughter. Pericardial, omental, scrotal and mesenteric fats were removed from hot carcass fat and weighed. After recording hot carcass weight, the carcasses were stored at 4°C for 24 h and weighted. The value was recorded as cold carcass weight. Both pre‐slaughter live weight and empty bodyweight (EBW) were used to calculate dressing percentage (DP1 and DP2, respectively). In order to calculate the EBW, the weight of the contents of the gastro‐intestinal tract was determined and subtracted from the PSW.

### 2.2 Determining Carcass Classification and Quality

EU regulation (European Commission 2017) classified the sheep in terms of age (as lambs or sheep) or weight (carcass weight below and above 13 kg) (Prache et al. 2022). Since the carcass weight of the lambs used in this study was greater than 13 kg, two photographs were taken of each carcass at a distance of 1.5 m using a Sony DCR‐SR190 digital camera, one is from the dorsal position and the other from the left position. Photographic documentation of all carcasses was completed, and then the carcasses were classified in terms of fatness and conformation. The reference point for carcass classification was the photographic standards described by the European Commission (European Commission 2017). A single experienced researcher was responsible for classifying all lamb carcasses in order to maintain consistency and accuracy in the classification process. Each CC and FC was subdivided as defined by Johansen et al. (2006) into three subclasses and then the final classification of carcasses was made according to a 1‐ to 15‐point scale.

Subsequent to the classification of the carcass, measurements of thoracic depth and carcass width were obtained in accordance with the methodologies described by Cañeque et al. (2004). Afterwards, the channel fat and kidney knob (KKCF) and tail were excised and weighed. Then, the carcasses were divided into two portions along the vertebral column. The length of the hind limbs and carcass internal length were then measured on the right side of carcass portion, in accordance with the methodology outlined by Velasco et al. (2000).

Backfat thicknesses were measured with callipers between the 12th‐13th thoracic vertebrae and third‐fourth lumbar vertebrae. Four measurements were implemented for each anatomical region, and the average of these was obtained as the fat thickness 12th–13th and fat thickness third‐fourth. The longissimus thoracis et lumborum muscle cross‐sectional area (LTSA) was measured between the 12th and 13th ribs by using millimetric paper. The left half of the carcass was jointed the seven cuts defined by the Akcapınar method (Akcapınar 1981), which included the neck, foreleg, shoulder, loin, hindlimb and other pieces (breast + flank). Each part was then entirely dissected into bone, fat (subcutaneous and intermuscular) and trimmed meat, including connective tissue and nerves. These were weighed separately. Proportions of each joint were calculated based on cold carcass weight. The sum of perinephric and pelvic, scrotal, pericardial, mesenteric and omental fats was called non‐carcass fat. The percentages of these fats were calculated in terms of the hot carcass weight. The combined weight of subcutaneous and intermuscular fats was recorded as carcass fat. To calculate total carcass muscle, bone and fat weight, muscle and bone weight was doubled. Weighing the combination of non‐carcass fat plus carcass fat and carcass fat weight was recorded as total body fat. Next, the carcass compactness index (cold carcass weight divided by the internal carcass length, g/cm), hind limb compactness index (hind limb weight divided by the hind limb length, g/cm) and chest roundness index (carcass width divided by thoracic depth) were calculated (Velasco et al. 2000).

### 2.3 Meat Sampling and Quality Analyses

The pH was measured postmortem at 24 h (pH_24_) by a portable pH meter (Testo 205, Testo AG, Lenzkirch, Germany) on different muscles: *Musculus longisimus thoracis et lumborum* (LTL) at the 12–13th thoracal vertebrae level, *Musculus semitendinosus* (ST) and *Musculus semimembranosus* (SM). Muscle samples for expressed juice and cooking loss analysis were collected from the fresh longissimus dorsi muscle sample. The meat colour parameters were measured from LTL, ST and SM on the carcass. For the calculation of meat‐expressed juice (%) values, 5 g meat pieces taken from the longissimus lumborum muscle were held under a pressure of 2250 g weight for 5 min, and the weight loss of the samples was determined (Beriain et al. 2000).

To obtain cooking loss, LTL muscle pieces were weighed, vacuumed and cooked in a polyethylene bag in a water bath at 80°C for 45 min. Afterward, samples were collected from the water bath and cooled under flowing water until the inside temperature has reached room temperature. All samples were stored for 24 h at 4°C. They were blotted dry with tissue paper then weighed. The difference between pre‐cooked and postcooked weight was divided by the pre‐cooked weight to obtain the cooking loss (%) (Honikel 1998).

Three colour measurements were carried out for each sample, and the average of these nine measurements was used to calculate the colour coordinates. The colour of the meat was assessed by means of the CIELAB colour space. Minolta CR 400 colour meter (Minolta Camera Co., Osaka, Japan) was used to determine L* (lightness), a* (redness) and b* (yellowness) values. Observation angle, aperture size and light source were set at 2°, 8 mm, and D65, respectively. The chromometer was calibrated using a standard white plate (*Y* = 93.8, *x* = 0.316, *y* = 0.3323).

Protein, moisture, ash, dry matter and intramuscular fat analysis were carried out by the AOAC method (Association of Official Analytical Chemists 1995). In these analyses, LTL muscle stored at −18 degrees was used.

### 2.4 Statistical Analysis

All data were analysed together for hierarchical cluster analysis in this study. In the first stage, hierarchic cluster analysis was performed on slaughter age, carcass 12–13th fat thickness, total carcass fat percentage, total noncarcass fat percentage, trimmed meat percentage, bone percentage, subcutan fat percentage, intermuscular fat percentage, tail fat percentage, KKCF percentage, LTSA, carcass compactness and hindquarter compactness, and chest roundness was analysed using the SPSS 21 software (IBM Inc., Chicago, II, United States) in order to classify the carcasses. Ward's hierarchical clustering method was performed in the present study. Next, the two clusters were compared for carcass characteristics and meat quality using an independent sample *T* test. In second stage, the effects of conformation and fatness class on carcass and meat quality characteristics were tested by GLM procedure, and slaughter age was added as a covariate.

## 3 Results

### 3.1 Cluster Analysis

For hierarchical cluster analysis, all data from three breeds were analysed together (Table 2). As a result of the analysis, two groups were obtained in terms of meat quality and carcass characteristics. One was the carcasses of Akkaraman and Herik with FT, and the other was the carcasses of Karayaka with TT.

**TABLE 2:** Mean values for some carcass and meat quality characteristics by clusters in lambs.

	Breeds by tail structure		
	Fat‐tailed breeds (Akkaraman and Herik breeds) (*n* = 30)	Thin‐tailed breed (Karayaka breed) (*n* = 15)	*p* value
Slaughter weight, kg	40.21 ± 0.14	40.11 ± 0.21	0.447
EBW, kg	34.72 ± 0.30	35.91 ± 0.24	0.113
Slaughter age, days	82.80 ± 4.93	101.27 ± 9.47	0.041
Fatness class	8.47 ± 0.20	8.53 ± 0.13	0.001
Conformation class	7.90 ± 0.15	8.47 ± 0.13	0.049
DP_1_, %	47.60 ± 0.38	43.86 ± 0.48	0.001
DP_2_, %	55.17 ± 0.44	48.99 ± 0.55	0.001
USG 3‐4 fat thickness, cm	4.26 ± 0.003	4.39 ± 0.003	0.151
USG 3‐4 muscle area, cm^2^	5.99 ± 0.18	6.62 ± 0.22	0.509
Cold tail fat proportion, %	15.21 ± 0.70	4.41 ± 0.29	0.001
KKCF, %	2.08 ± 0.20	3.46 ± 0.26	0.738
Carcass L3‐4 fat thickness, cm	3.67 ± 0.39	5.40 ± 0.41	0.168
Carcass 12‐13 fat thickness, cm	3.10 ± 0.30	3.71 ± 0.18	0.312
LTSA, cm^2^	11.59 ± 0.31	12.11 ± 0.41	0.937
Neck, %	6.44 ± 0.14	7.83 ± 0.16	0.226
Shoulder, %	7.86 ± 0.26	9.59 ± 0.21	0.301
Loin, %	5.80 ± 0.15	7.55 ± 0.15	0.027
Hind limb, %	29.56 ± 0.82	32.17 ± 0.62	0.042
Foreleg, %	15.45 ± 0.15	16.84 ± 0.23	0.699
Other pieces, %	18.94 ± 0.22	19.80 ± 0.28	0.710
Carcass compactness index, g/cm	353.06 ± 4.73	313.43 ± 5.40	0.265
Chest roundness index	0.84 ± 0.03	0.70 ± 0.03	0.537
Hindlimb compactness index	70.54 ± 0.94	73.21 ± 1.88	0.234
Trimmed meat, %	49.21 ± 0.80	54.90 ± 1.00	0.708
Bone, %	17.38 ± 0.34	16.90 ± 0.39	0.143
Subcutan fat, %	11.70 ± 0.73	15.68 ± 0.77	0.094
Intermuscular fat, %	4.64 ± 0.26	7.42 ± 0.30	0.312
Total carcass fat, %	15.96 ± 1.44	23.24 ± 0.86	0.005
Total noncarcass fat, %	22.68 ± 0.55	9.81 ± 0.52	0.001
Muscle/bone ratio	2.85 ± 0.05	3.27 ± 0.09	0.222
Muscle/total fat ratio	1.38 ± 0.07	1.67 ± 0.09	0.596
Muscle/carcass fat ratio	3.46 ± 0.32	2.44 ± 0.13	0.011
Musculus longisimus thoracis et lumborum			
pH_24_	5.58 ± 0.02	5.60 ± 0.03	0.661
L*_24_	40.76 ± 0.90	38.23 ± 0.70	0.030
a*_24_	14.85 ± 0.32	16.44 ± 0.57	0.194
b*_24_	6.22 ± 0.25	6.92 ± 0.42	0.610
Cooking loss, %	33.22 ± 0.39	29.95 ± 0.57	0.280
Expressed juice, %	8.83 ± 1.06	9.12 ± 0.51	0.029
Dry matter, %	25.54 ± 0.22	27.20 ± 0.31	0.990
Crude ash, %	1.34 ± 0.05	1.32 ± 0.08	0.569
Crude fat, %	2.80 ± 0.10	4.03 ± 0.16	0.311
Crude protein, %	20.93 ± 0.19	20.38 ± 0.30	0.982

*Note*: DP_1_: Dressing percentage according to the preslaughter weight. DP_2_: Dressing percentage according to the empty body weight.

Slaughter age, fatness class, conformation class, loin percentage, hind limb percentage and total carcass fat percentage of TT breed were higher than in FT breeds. In addition, DP_1_, DP_2,_ cold tail fat percentage and total noncarcass fat percentage of FT breeds were higher than in TT breed. Besides, while L*_24_ value was higher in FT breeds, expressed juice value was higher in TT breed.

### 3.2 EUROP Fatness Class and Carcass Characteristics

Carcass characteristics of FT and TT lambs are presented in Tables 3 and 4 in terms of FC. As the European fat class increased in FT lambs, USG 3–4 fat thickness, scrotal fat percentage and carcass 3–4 fat thickness gradually increased. No significant difference was found (*p* > 0.05) between the carcass joint proportions as the FC increased. As fat class increased, total carcass fat percentage, subcutaneous fat percentage and intermuscular fat percentage increased, while total noncarcass fat percentage, trimmed meat percentage, bone percentage, muscle/total fat ratio and muscle/carcass fat ratio decreased.

**TABLE 3:** Effect of EUROP fatness class on carcass characteristics in fat‐tailed lambs.

	EUROP fatness class				
	7 (*n* = 10)	8 (*n* = 11)	9 (*n* = 9)	SEM	P Value
Slaughter weight, kg	39.87	40.72	40.07	0.130	0.094
EBW, kg	34.02	34.65	35.52	0.299	0.410
Hot carcass weight, kg	18.92	18.88	19.28	0.140	0.327
Cold carcass weight, kg	18.35	18.30	18.73	0.138	0.313
Shrinkage, %	3.03	3.04	2.85	0.064	0.713
DP_1_, %	47.46	46.37	48.11	0.365	0.119
DP_2_, %	55.69	54.55	54.31	0.437	0.351
USG 3–4 fat thickness, cm	3.12^b^	4.1^ab^	5.10^a^	0.023	0.018
USG 3–4 muscle area, cm^2^	6.23	5.72	6.01	0.185	0.761
USG 3–4 muscle thickness, cm	1.83	1.77	1.73	0.031	0.707
USG 3–4 muscle width, cm	4.37	3.96	4.30	0.082	0.203
Hot tail fat proportion, %	14.66	14.01	13.93	0.863	0.913
Cold tail fat proportion, %	14.44	14.18	16.76	0.710	0.543
Pelvic fat, %	0.39	0.61	0.43	0.037	0.169
Perinephric fat, %	0.32	0.48	0.93	0.137	0.387
KKCF, %	1.58	2.67	2.15	0.191	0.183
Pericardial fat, %	0.14	0.20	0.21	0.015	0.257
Scrotal fat, %	0.72^b^	0.91^ab^	1.21^a^	0.074	0.040
Omental fat, %	0.82	1.49	1.29	0.136	0.253
Carcass L3–4 fat thickness, cm	2.33^b^	3.45^ab^	4.57^a^	0.359	0.050
Carcass 12–13 fat thickness, cm	3.14	3.41	2.22	0.300	0.436
LTSA, cm^2^	12.70	11.57	10.53	0.297	0.125
Neck, %	6.39	6.38	6.43	0.145	0.975
Shoulder, %	7.36	7.94	8.44	0.265	0.554
Loin, %	5.66	5.94	6.14	0.153	0.422
Hind limb, %	30.74	30.29	29.80	0.309	0.771
Foreleg, %	16.02	15.58	15.21	0.139	0.078
Other pieces, %	18.54	18.97	18.92	0.228	0.726
Carcass compactness index, g/cm	340.93	353.84	363.43	4.790	0.465
Chest roundness index	0.76	0.81	0.92	0.024	0.101
Hindlimb compactness index	68.48	68.84	72.32	0.916	0.212
Trimmed meat, %	53.62^a^	50.09^ab^	46.34^b^	0.631	0.001
Bone, %	18.64^a^	17.90^a^	15.45^b^	0.282	0.004
Subcutan fat, %	8.01^b^	11.09^ab^	12.66^a^	0.592	0.003
Intermuscular fat, %	3.45^b^	4.88^ab^	5.38^a^	0.244	0.044
Total carcass fat, %	11.41^b^	15.96^ab^	18.05^a^	1.344	0.037
Total noncarcass fat, %	22.78^a^	21.00^ab^	17.57^b^	0.461	0.005
Muscle/bone ratio	2.88	2.82	2.68	0.045	0.099
Muscle/total fat ratio	1.81^a^	1.39^ab^	1.18^b^	0.056	0.001
Muscle/carcass fat ratio	5.46^a^	3.24^b^	2.91^b^	0.248	0.001

*Note*: DP_1_: Dressing percentage according to the preslaughter weight. DP_2_: Dressing percentage according to the empty body weight.

**TABLE 4:** Effect of EUROP fatness class on carcass characteristics in thin‐tailed lambs.

	EUROP fatness class		
	8 (*n* = 8)	9 (*n* = 7)	*p* value
Slaughter weight, kg	40.40 ± 0.37	39.85 ± 0.22	0.207
EBW, kg	35.80 ± 0.46	36.00 ± 0.23	0.678
Hot carcass weight, kg	17.26 ± 0.19	17.87 ± 0.19	0.776
Cold carcass weight, kg	16.77 ± 0.19	17.32 ± 0.25	0.659
Shrinkage, %	2.90 ± 0.15	3.17 ± 0.50	0.631
DP_1_, %	42.74 ± 0.66	44.84 ± 0.50	0.547
DP_2_, %	48.26 ± 0.86	49.64 ± 0.68	0.547
USG 3–4 fat thickness, cm	0.42 ± 0.05	0.45 ± 0.02	0.577
USG 3–4 muscle area, cm^2^	6.44 ± 0.31	6.77 ± 0.32	0.488
USG 3–4 muscle thickness, cm	1.89 ± 0.07	1.90 ± 0.05	0.809
USG 3–4 muscle width, cm	4.02 ± 0.15	4.18 ± 0.11	0.396
Hot tail fat proportion, %	3.90 ± 0.37	4.81 ± 0.39	0.122
Cold tail fat proportion, %	3.92 ± 0.37	4.84 ± 0.39	0.114
Pelvic fat, %	0.75 ± 0.09	0.82 ± 0.10	0.599
Perinephric fat, %	0.52 ± 0.07	0.73 ± 0.12	0.173
KKCF, %	3.03 ± 0.30	3.83 ± 0.39	0.135
Pericardial fat, %	0.16 ± 0.01	0.22 ± 0.02	0.064
Scrotal fat, %	1.19 ± 0.13^b^	1.50 ± 0.15^a^	0.047
Omental fat, %	1.68 ± 0.21	2.17 ± 0.24	0.157
Carcass L3–4 fat thickness, cm	5.52 ± 0.42	5.30 ± 0.69	0.805
Carcass 12–13 fat thickness, cm	3.54 ± 0.26	3.86 ± 0.26	0.400
LTSA, cm^2^	11.17 ± 0.46^b^	12.93 ± 0.52^a^	0.026
Neck, %	8.02 ± 0.27	7.66 ± 0.17	0.270
Shoulder, %	9.95 ± 0.28	9.27 ± 0.29	0.116
Loin, %	7.50 ± 0.11	7.60 ± 0.28	0.760
Hind limb, %	31.84 ± 0.82	32.46 ± 0.96	0.639
Foreleg, %	17.02 ± 0.31	16.69 ± 0.35	0.506
Other pieces, %	20.50 ± 0.29	20.06 ± 0.46	0.336
Carcass compactness index, g/cm	302.25 ± 2.02^b^	323.21 ± 8.77^a^	0.048
Chest roundness index	0.88 ± 0.06	0.80 ± 0.02	0.238
Hindlimb compactness index	69.77 ± 1.93	76.22 ± 2.78	0.087
Trimmed meat, %	57.40 ± 0.75^a^	52.71 ± 1.38^b^	0.013
Bone, %	17.00 ± 0.75	16.81 ± 1.03	0.821
Subcutan fat, %	13.87 ± 0.65^b^	17.27 ± 1.06^a^	0.021
Intermuscular fat, %	7.14 ± 0.33	7.65 ± 0.49	0.415
Total carcass fat, %	19.95 ± 1.20^b^	24.94 ± 1.28^a^	0.024
Total noncarcass fat, %	9.46 ± 0.77	10.04 ± 0.73	0.652
Muscle/bone ratio	3.14 ± 0.09	3.41 ± 0.15	0.136
Muscle/total fat ratio	1.89 ± 0.11^a^	1.47 ± 0.11^b^	0.018
Muscle/carcass fat ratio	2.77 ± 0.14^a^	2.16 ± 0.15^b^	0.012

*Note*: DP_1_: Dressing percentage according to the preslaughter weight. DP_2_: Dressing percentage according to the empty body weight.

In TT lambs, as the European FC increased, carcass compactness index, scrotal fat percentage, subcutaneous fat percentage and LTSA increased, while trimmed meat percentage, muscle/total fat ratio and muscle/carcass fat ratio decreased.

### 3.3 EUROP Fatness Class and Meat Quality Characteristics

Meat quality characteristics of FT and TT lambs are presented Tables 5 and 6 in terms of FC. In FT lambs, the effect of FC on meat quality traits and chemical properties of three different muscles was not significant (*p* > 0.05). Besides, in TT lambs, there was a decrease in pH_24_ of ST muscle, while there was an increase in expressed juice, redness and yellowness values of LTL muscle with the increase of FC from 8 to 9.

**TABLE 5:** Effect of EUROP fatness class on meat quality characteristics in fat‐tailed lambs.

	EUROP fatness class				
	7 (*n* = 10)	8 (*n* = 11)	9 (*n* = 9)	SEM	*p* value
Musculus longisimus thoracis et lumborum					
pH_24_	5.51	5.58	5.57	0.024	1.193
L*_24_	38.51	42.97	40.86	0.902	0.347
a*_24_	14.36	14.84	15.00	0.332	0.849
b*_24_	5.58	6.63	6.28	0.258	0.535
Cooking loss, %	32.91	32.77	33.80	0.409	0.778
Expressed juice, %	8.13	9.06	9.02	0.527	0.587
Dry matter, %	25.27	25.70	25.57	0.234	0.925
Crude ash, %	1.21	1.34	1.41	0.056	0.577
Crude fat, %	2.63	2.64	3.02	0.100	0.378
Crude protein, %	20.89	20.37	21.26	0.189	0.235
Musculus semitendinosus					
pH_24_	5.65	5.56	5.59	0.017	0.178
L*_24_	49.19	48.99	50.17	0.533	0.871
a*_24_	14.62	13.24	13.94	0.510	0.802
b*_24_	7.94	7.50	8.06	0.219	0.633
Musculus semimembranosus					
pH_24_	5.53	5.49	5.50	0.015	0.775
L*_24_	40.01	41.86	41.07	0.634	0.600
a*_24_	16.97	18.03	18.05	0.583	0.904
b*_24_	7.80	8.52	8.40	0.286	0.746

**TABLE 6:** Effect of EUROP fatness class on meat quality characteristics in thin‐tailed lambs.

	EUROP fatness class		
	8 (*n* = 8)	9 (*n* = 7)	*p* value
Musculus longisimus thoracis et lumborum			
pH_24_	5.65 ± 0.05	5.54 ± 0.04	0.103
L*_24_	37.04 ± 1,27	39.26 ± 0.57	0.120
a*_24_	15.12 ± 0.65	17.60 ± 0.69	0.023
b*_24_	5.78 ± 0.52	7.92 ± 0.40	0.005
Cooking loss, %	29.14 ± 0.73	30.65 ± 0.83	0.199
Expressed juice, %	5.88 ± 0.49	11.42 ± 1.42	0.004
Dry matter, %	27.30 ± 0.41	27.98 ± 0.22	0.633
Crude ash, %	1.34 ± 0.13	1.29 ± 0.10	0.741
Crude fat, %	3.78 ± 0.24	4.25 ± 0.20	0.157
Crude protein, %	20.21 ± 0.49	20.53 ± 0.38	0.613
Musculus semitendinosus			
pH_24_	5.64 ± 0.01	5.52 ± 0.04	0.026
L*_24_	45.25 ± 0.70	46.96 ± 0.78	0.130
a*_24_	14.65 ± 0.36	15.37 ± 0.68	0.048
b*_24_	7.48 ± 0.30	7.78 ± 0.46	0.074
Musculus semimembranosus			
pH_24_	5.55 ± 0.01	5.53 ± 0.03	0.548
L*_24_	38.53 ± 1.14	39.35 ± 1.03	0.601
a*_24_	17.42 ± 1.53	19.69 ± 1.04	0.232
b*_24_	7.63 ± 0.63	8.25 ± 0.36	0.396

### 3.4 EUROP Conformation Class and Carcass Characteristics

Carcass characteristics of FT and TT lambs are presented in Tables 7 and 8 in terms of CC. In FT lambs, scrotal fat percentage, carcass 3–4 fat thickness and hindlimb compactness index increased as the European CC increased. In addition, the lowest subcutaneous fat and intermuscular fat percentage, the highest bone and trimmed meat percentages and muscle/total fat and muscle/carcass fat ratio were found in carcasses of CC 7. In TT lambs, the effect of CC on ultrasonic measurements, carcass indexes, carcass muscle, bone and fat percentages was not generally significant.

**TABLE 7:** Effect of EUROP conformation class on carcass characteristics in fat‐tailed lambs.

	EUROP conformation class				
	7 (*n* = 12)	8 (*n* = 9)	9 (*n* = 9)	SEM	*p* value
Slaughter weight, kg	40.36	40.24	39.99	0.141	0.511
EBW, kg	34.04	34.82	35.52	0.287	0.115
Hot carcass weight, kg	18.75	19.51	19.25	0.133	0.069
Cold carcass weight, kg	18.16	18.94	18.71	0.130	0.068
Shrinkage, %	3.15	2.89	2.83	0.058	0.061
DP_1_, %	46.49	48.49	48.17	0.360	0.054
DP_2_, %	55.19	56.07	54.24	0.436	0.277
USG 3–4 fat thickness, cm	0.37	0.44	0.49	0.026	0.191
USG 3–4 muscle area, cm^2^	5.91	5.83	6.28	0.183	0.597
USG 3–4 muscle thickness, cm	1.81	1.77	1.73	0.030	0.535
USG 3–4 muscle width, cm	4.29	4.07	4.03	0.085	0.368
Hot tail fat proportion, %	14.66	16.70	15.05	0.783	0.385
Cold tail fat proportion, %	14.66	16.96	15.20	0.692	0.258
Pelvic fat, %	0.40	0.53	0.56	0.038	0.209
Perinephric fat, %	0.34	0.36	0.87	0.135	0.225
KKCF, %	1.80	1.99	2.53	0.198	0.305
Pericardial fat, %	0.17	0.15	0.22	0.015	0.203
Scrotal fat, %	0.83^b^	1.06^ab^	1.31^a^	0.076	0.044
Omental fat, %	0.99	1.05	1.47	0.139	0.338
Carcass L3‐4 fat thickness, cm	2.60^a^	3.86^ab^	4.90^b^	0.364	0.042
Carcass 12‐13 fat thickness, cm	2.36	3.85	3.32	0.286	0.103
LTSA, cm^2^	12.26	11.50	10.78	0.303	0.140
Neck, %	6.32	6.16	6.87	0.132	0.103
Shoulder, %	7.88	7.57	8.10	0.268	0.741
Loin, %	5.61	5.58	6.28	0.146	0.116
Hind limb, %	30.60	29.87	30.12	0.304	0.586
Foreleg, %	15.75	15.49	15.03	0.145	0.138
Other pieces, %	17.86^ab^	18.34^a^	16.63^b^	0.208	0.045
Carcass compactness index, g/cm	344.64	362.63	354.72	4.728	0.291
Chest roundness index	0.84	0.83	0.85	0.027	0.935
Hindlimb compactness index	67.85^b^	69.99^ab^	73.94^a^	0.864	0.032
Trimmed meat, %	51.96^a^	47.17^b^	47.60^b^	0.714	0.014
Bone, %	18.89^a^	16.25^b^	16.50^b^	0.264	0.001
Subcutan fat, %	8.77^a^	13.42^b^	13.89^b^	0.605	0.002
Intermuscular fat, %	3.94^a^	4.43^ab^	5.78^b^	0.230	0.008
Total carcass fat, %	12.28	17.44	19.36	1.357	0.060
Total noncarcass fat, %	22.16	19.94	19.46	0.527	0.084
Muscle/bone ratio	2.76	2.91	2.89	0.048	0.367
Muscle/total fat ratio	1.67^a^	1.21^b^	1.16^b^	0.059	0.001
Muscle/carcass fat ratio	4.64^a^	2.86^b^	2.49^b^	0.273	0.005

*Note*: DP_1_: Dressing percentage according to the preslaughter weight. DP_2_: Dressing percentage according to the empty body weight.

**TABLE 8:** Effect of EUROP conformation class on carcass characteristics in thin‐tailed lambs.

	EUROP conformation class		
	8 (*n* = 8)	9 (*n* = 7)	*p* value
Slaughter weight, kg	40.19 ± 0.26	40.01 ± 0.37	0.700
EBW, kg	35.92 ± 0.36	35.90 ± 0.34	0.971
Hot carcass weight, kg	17.53 ± 0.23	17.64 ± 0.21	0.749
Cold carcass weight, kg	17.06 ± 0.23	17.08 ± 0.28	0.960
Shrinkage, %	2.79 ± 0.16	3.33 ± 0.55	0.341
DP_1_, %	43.65 ± 0.75	44.09 ± 0.63	0.665
DP_2_, %	48.86 ± 0.93	49.14 ± 0.62	0.812
USG 3–4 fat thickness, cm	0.45 ± 0.04	0.43 ± 0.04	0.816
USG 3–4 muscle area, cm^2^	6.40 ± 0.27	6.87 ± 0.35	0.305
USG 3–4 muscle thickness, cm	1.88 ± 0.07	1.92 ± 0.04	0.604
USG 3–4 muscle width, cm	4.04 ± 0.13	4.18 ± 0.13	0.444
Hot tail fat proportion, %	4.20 ± 0.37	4.61 ± 0.47	0.502
Cold tail fat proportion, %	4.21 ± 0.37	4.64 ± 0.47	0.485
Pelvic fat, %	0.77 ± 0.08	0.80 ± 0.11	0.826
Perinephric fat, %	0.59 ± 0.06	0.68 ± 0.15	0.578
KKCF, %	3.43 ± 0.33	3.50 ± 0.45	0.902
Pericardial fat, %	0.18 ± 0.02	0.20 ± 0.03	0.531
Scrotal fat, %	1.19 ± 0.10	1.54 ± 0.18	0.041
Omental fat, %	1.97 ± 0.23	1.91 ± 0.27	0.861
Carcass L3–4 fat thickness, cm	5.25 ± 0.30	5.58 ± 0.84	0.706
Carcass 12–13 fat thickness, cm	3.81 ± 0.30	3.59 ± 0.21	0.569
LTSA, cm^2^	11.99 ± 0.57	12.24 ± 0.63	0.773
Neck, %	7.86 ± 0.18	7.79 ± 0.28	0.834
Shoulder, %	9.92 ± 0.23	9.21 ± 0.34	0.100
Loin, %	7.67 ± 0.17	7.41 ± 0.27	0.412
Hind limb, %	31.71 ± 0.69	32.71 ± 1.10	0.443
Foreleg, %	16.73 ± 0.29	16.98 ± 0.39	0.608
Other pieces, %	29.91 ± 0.51	29.67 ± 0.21	0.685
Carcass compactness index, g/cm	312.86 ± 7.15	314.08 ± 8.79	0.915
Chest roundness index	0.87 ± 0.06	0.80 ± 0.02	0.306
Hindlimb compactness index	73.21 ± 2.58	73.21 ± 2.98	0.995
Trimmed meat, %	55.92 ± 1.19	53.73 ± 1.65	0.295
Bone, %	17.02 ± 0.42	16.76 ± 0.71	0.753
Subcutan fat, %	14.83 ± 0.69	16.66 ± 1.42	0.250
Intermuscular fat, %	7.36 ± 0.51	7.48 ± 0.32	0.850
Total carcass fat, %	22.24 ± 1.18	23.23 ± 3.55	0.998
Total noncarcass fat, %	9.83 ± 0.65	9.78 ± 0.88	0.958
Muscle/bone ratio	3.30 ± 0.10	3.24 ± 0.16	0.746
Muscle/total fat ratio	1.75 ± 0.12	1.57 ± 0.14	0.344
Muscle/carcass fat ratio	2.58 ± 0.17	2.29 ± 0.19	0.280

*Note*: DP_1_: Dressing percentage according to the preslaughter weight. DP_2_: Dressing percentage according to the empty body weight.

### 3.5 EUROP Conformation Class and Meat Quality Characteristics

Meat quality characteristics of FT and TT lambs are presented Tables 9 and 10 in terms of CC. No significant effect of European CC was found on meat quality and chemical properties of three different muscles in FT lambs (*p* > 0.05). Besides, in TT lambs, the effect of CC was not significant on meat quality and chemical properties.

**TABLE 9:** Effect of EUROP conformation class on meat quality characteristics in fat‐tailed lambs.

	EUROP conformation class				
	7 (*n* = 12)	8 (*n* = 9)	9 (*n* = 9)	SEM	*p* value
Musculus longisimus thoracis et lumborum					
pH_24_	5.58	5.59	5.59	0.026	0.976
L*_24_	40.52	41.80	40.04	0.935	0.747
a*_24_	14.45	14.94	15.28	0.325	0.563
b*_24_	6.10	6.23	6.38	0.264	0.908
Cooking loss, %	33.71	32.40	33.38	0.397	0.389
Expressed juice, %	8.56	9.67	9.31	0.530	0.665
Dry matter, %	25.11	26.11	25.56	0.218	0.177
Crude ash, %	1.30	1.24	1.51	0.053	0.127
Crude fat, %	2.64	2.90	2.91	0.101	0.431
Crude protein, %	20.65	21.29	20.94	0.195	0.399
Musculus semitendinosus					
pH_24_	5.58	5.62	5.55	0.018	0.253
L*_24_	50.00	48.86	49.22	0.523	0.646
a*_24_	13.45	13.42	14.76	0.498	0.482
b*_24_	7.79	7.49	8.49	0.209	0.177
Musculus semimembranosus					
pH_24_	5.50	5.50	5.52	0.015	0.825
L*_24_	40.82	41.31	40.11	0.640	0.763
a*_24_	17.49	17.74	18.12	0.578	0.902
b*_24_	8.14	7.51	8.89	0.269	0.151

**TABLE 10:** Effect of EUROP conformation class on meat quality characteristics in thin‐tailed lambs.

	EUROP conformation class		
	8 (*n* = 8)	9 (*n* = 7)	*p* value
Musculus longisimus thoracis et lumborum			
pH_24_	5.61 ± 0.04	5.58 ± 0.06	0.586
L*_24_	37.68 ± 1.23	38.85 ± 0.59	0.428
a*_24_	16.82 ± 0.71	16.01 ± 0.94	0.494
b*_24_	6.96 ± 0.69	6.88 ± 0.50	0.927
Cooking loss, %	30.47 ± 0.80	29.35 ± 0.82	0.347
Expressed juice, %	8.46 ± 1.35	9.26 ± 1.77	0.720
Dry matter, %	27.58 ± 0.43	27.90 ± 0.29	0.729
Crude ash, %	1.27 ± 0.12	1.37 ± 0.09	0.503
Crude fat, %	3.95 ± 0.26	4.12 ± 0.20	0.614
Crude protein, %	20.07 ± 0.30	20.73 ± 0.54	0.288
Musculus semitendinosus			
pH_24_	5.61 ± 0.02	5.54 ± 0.06	0.307
L*_24_	46.34 ± 0.56	45.95 ± 1.06	0.739
a*_24_	14.63 ± 0.60	15.50 ± 0.49	0.289
b*_24_	7.45 ± 0.37	7.86 ± 0.43	0.485
Musculus semimembranosus			
pH_24_	5.54 ± 0.01	5.55 ± 0.03	0.876
L*_24_	38.64 ± 1.16	39.34 ± 0.97	0.657
a*_24_	18.59 ± 1.63	18.68 ± 0.84	0.962
b*_24_	7.84 ± 0.52	8.10 ± 0.49	0.727

## 4 Discussion

### 4.1 EUROP Fatness Class and Carcass Characteristics

It was determined that when the FC increased from 8 to 9 in TT lamb carcasses, carcass compactness index, carcass LTSA and subcutaneous fat percentage increased, while trimmed meat percentage, muscle/carcass fat ratio and muscle/total fat ratio decreased. Besides, in this study, the scrotal fat percentage increased as the FC increased in both FT and TT lamb carcasses. Velasco et al. (2000) reported that fatness increased significantly as lamb live weight increased from 10 to 12 kg. The authors also reported that the development of adipose tissue had begun in these lambs, although they were still very young. Jeremiah et al. (1997) reported that the thickness of subcutaneous fat increased as carcass fatness increased. Miguel et al. (2003) determined that even for a small weight range, differences in fatness based on carcass weight are very important, as they influence carcass classification and carcass price at the abattoir. Díaz et al. (2003) found that as weight increased, only the proportion of bone tissue decreased. Díaz et al. (2003) and Janicki et al. (2000) found that as FC increased the thickness of backfat on the longissimus muscle and on the rib increased, the proportion of adipose tissue increased and the proportion of muscle and bone in the carcass decreased, but the effect of FC on cross‐sectional area of Musculus Longisimus dorsi was not significant. Besides, Stanisz et al. (2012) determined that there were no significant differences between the FCs for any of the quality traits or the rib‐eye area. M/B and M/TF supply useful information about carcass tissue composition. Carcasses with a high M/B ratio are more commercially viable and in greater demand from consumers. Mahgoubt and Lodge (1994) and Velasco et al. (2000) found that the M/B ratio increases with carcass weight and besides that the amount of lean tissue increases faster than bone tissue as carcass weight increases. The authors stated that the fat proportion increased with age, while the bone proportion decreases. In the presented study, differences in M/B ratio among fatness classes were not significant in both FT and TT lamb carcasses.

Ekiz et al. (2024) reported that the subcutaneous fat percentage increased and the muscle/carcass fat ratio decreased as the FC increased in Kivircik lamb (TT) carcasses. Besides, the M/TF ratio in TT lambs decreased when the fatness class increased from 8 to 9 in the present study. These results indicate that bone tissue develops earlier than muscle tissue, while fat tissue develops later. Unlike our study findings, Stanisz et al. (2012) stated that as fatness increased, the proportion of leg with shank and forearm with carpus decreased, while the proportion of saddle, rack and flank with ribs and sternum increased; therefore, the authors conclude that the percentage of joints in the carcass was highly significantly influenced by the degree of fatness. In present study, it was found that as the FC increased in FT lamb carcasses, total carcass fat percentage, subcutaneous fat percentage and intermuscular fat percentage increased, while total non‐carcass fat percentage, trimmed meat percentage, bone percentage, muscle/total fat ratio and muscle/carcass fat ratio decreased. Ekiz et al. (2024) reported that as the FC increases in Akkaraman lamb (FT) carcasses, the total carcass fat percentage and subcutaneous fat percentage obtained from hind limb tissue dissection increased, while the bone percentage and muscle/carcass fat ratio decreased. It can be concluded that the results of the present study for carcass composition are consistent with the above mentioned reports.

### 4.2 EUROP Fatness Class and Meat Quality Characteristics

It was determined that as the FC increased from 8 to 9 in TT lamb carcasses, the expressed juice, the a*_24_ and b*_24_ values ​​of the LTL increased, and the pH_24_ value of the ST muscle decreased in the present study. On the other hand, in FT carcasses, the effect of FC on meat quality traits and chemical properties of the three different muscles was insignificant. Colour, pH, expressed juice and cooking loss are important meat quality characteristics. They can influence consumer preferences. To turn muscle into flesh, various biochemical processes and changes take place at the cellular level. Rigor mortis is an essential stage in this process. This is associated with changes in the myofibrils of the muscles. ATP and glycogen reserves are also expected to drop, causing the muscle pH to drop from 7.0 to less than 6.0 (Ouali et al. 2006; Pearce et al. 2011). A level of 5.8 or less is recommended 24 h after slaughter in order to avoid problems with meat quality. Meat shelf life, colour, tenderness, flavour and juiciness depend on the final pH of the meat. The quality of meat can be affected by an abnormal pH, particularly in terms of its colour and tenderness (Tejeda et al. 2008; Teke et al. 2014). All lambs were kept under similar pre‐slaughter conditions in the current study. There was no significant difference in pH between the fat classes in the present study. The pH values were similar in all classes. In present study, most values were within pH_24_ 5.60 and 5.84 (Okeudo and Moss 2005). Of all the carcasses, only one had a pH above 6.0. This suggests that animals of different fatness have the same susceptibility to stress and the same muscle glycogen content. These results are in line with those of Kadim et al. (1993).

Meat colour is an important factor influencing consumer choice. Colour is affected by the myoglobin state in the meat. The concentration of desoxymyoglobin molecules is higher in freshly cut meat. It makes it reddish‐purple. However, when the desoxymyoglobin is exposed to oxygen, it is converted to oxymyoglobin, which gives the desired bright red colour. Finally, with continued exposure, as oxymyoglobin oxidises to metamyoglobin, the meat turns brown (Moore et al. 2003). Meat colour can make or break a decision to buy, both through its intensity and any instability or change in colour. The myoglobin content and pH of fresh meat determine its colour (Berthelot et al. 2012). Sanudo et al. (2000) found that FC had a significant influence on meat redness, yellowness and lightness values. The authors stated that as the FC increased, these colour parameters also increased in light lambs. Dubeski et al. (1997) reported that there was no significant difference in meat lightness between fatness classes in heifers and concluded that fatness had little influence on meat lightness. In a study conducted on suckling lambs with very small slaughter weight differences, Díaz et al. (2003) found that as the slaughter weight increased, the L* and b* values decreased and a* value increased, particularly in the rectus abdominis muscle. In another study on suckling lambs, Sanudo et al. (2000) found that significant differences between FCs in terms of meat redness and yellowness values, and they observed that the meat of the lamb exhibits a darkening in colour as the fatness class increases. The findings of the present study for meat colour parameters are also consistent with these reports.

Nogalski et al. (2019) found that the fat cover had a beneficial influence on the water‐holding capacity and tenderness of meat in young bulls. Yalcıntan et al. (2024) found that no significant difference among FCs with regard to expressed juice, but there was a tendency to increase expressed juice value as FC increases in goat kids. Ekiz et al. (2024) reported that expressed juice values increased as carcass fatness increased in the Kivircik breed. In the present study, It was determined that the expressed juice value increased as the FC increased from 8 to 9 in TT lamb carcasses. One possible explanation for the observed increase in expressed juice with increasing fatness is that adipose tissue may protect the meat against moisture loss. During the storage period, some of the humidity content within the muscle fibres is lost through evaporation. Nevertheless, the presence of fat can obstruct the evaporation of moisture by acting as a mechanical obstacle, thereby diminishing the evaporation rate (Tichenor 1969).

Lamb meat contains high content of protein, vitamin B_12_, essential amino acids and essential fatty acids (Li et al. 2022). The chemical composition of meat is relatively constant (about 75% of water, 19%–25% of proteins, 3%–5% intramuscular fat, and 1%–2% of minerals and glycogen) (Hocquette et al. 2010). Ciliberti et al. (2021) found that the chemical composition ranged from 21.55% to 22.20% for protein and 0.76% to 1.77% for intramuscular lipid content in five sheep breeds reared in Italy. Aksoy and Ulutaş (2016) reported that protein, intramuscular fat and ash of Longissimus lumborum concerning slaughtered at 40 kg Karayaka lambs were 20.13%, 2.41% and 1.07%, respectively. Ugurlu et al. (2017) reported that the same values ​​for FT Herik lambs slaughtered at the same weight were 22.21, 2.98, and 1.02, respectively. The results of the current study for the protein and ash values ​​obtained from FT carcasses in FC and CCs were compatible with these ​​reported above. However, in the present study, it was found that the intramuscular fat value obtained from FT carcasses was higher than the literature values. However, since these values ​​obtained from both FT and TT carcasses were less than 5%, the meats can be categorised as lean meat by committee (Food Advisory Committee 1990) criteria, but FC 9 approached this limit (4.25%) in TT lamb.

Ekiz et al. (2024) determined that there was no significant difference in meat quality parameters as the FC increased in FT Akkaraman lamb carcasses. The results of the current study on meat quality traits in FT carcasses are consistent with the research findings of Ekiz et al. (2024). In present study, it was found that the European fatness classification system could distinguish Karayaka carcasses in terms of meat colour and water‐holding capacity. However, in FT lamb carcasses, the European FC did not have the ability to distinguish carcasses with regard to meat quality characteristics.

### 4.3 EUROP Conformation Class and Carcass Characteristics

In the European Union, carcass weight, carcass fatness and carcass conformation are used as quality criteria in the approved system for the classification of sheep carcasses. In FT and TT carcass, the effect of CC ​​on carcass compactness index and chest roundness index was insignificant. Besides, the difference between CCs in TT lambs was insignificant, while the trimmed meat percentage decreased as the number of classes increased in FT lambs. Janiszewski et al. (2018) determined that there were significant differences in carcass traits among conformations. The higher the carcass weight, the higher the CC. No significant differences were found between the fat classes 1–3 for any of the quality traits and also for the rib eye area. The results of the current study for LTSA are also in accordance with these reports.

Short and broad carcasses look more compact, and this is apparently favoured by consumers as they prefer cuts with more muscle (Texeira et al. 2004). A common finding in the best conformation carcasses is a relatively high muscle/bone ratio. Kempster et al. (1981) stated that conformation is not a reliable indicator of the composition of the carcass. However, Taylor et al. (1989) stated that fatness may be a better indicator of carcass composition because of the inverse relationship between fat and muscle. Similarly, Miguel et al. (2003) found that carcass conformation is usually a secondary quality criterion, implicitly included in the fatness score. In the present study, it was found that the effect of CC on the trimmed meat and bone ratio was significant in FT carcasses and that there was no difference in these parameters between CCs in TT carcasses. Ekiz et al. (2024) stated that the effect of CC on the total fat percentage, muscle/bone ratio and muscle/fat ratio was insignificant in TT carcasses. The findings of the present study are compatible with the research results of Ekiz et al. (2024).

In the latter study, it was reported that as the CC increases in Akkaraman breed, there is no significant difference between the joint percentages except the shoulder percentage. In the present study, there is no significant difference between the joint percentages except the other pieces percentage in FT lambs. In the present study, it was noteworthy that similar to the findings in the FC, as the CC increased, the scrotal fat percentage increased in both FT and TT lambs.

When the European conformation system was compared in terms of distinguishing carcasses of FT and TT lambs slaughtered at the same live weight, it was determined that it was successful in terms of trimmed meat percentage and bone percentage in FT lamb carcasses, but not in TT lamb carcasses. In terms of carcass characteristics, it was determined that the European fatness classification system was more successful in distinguishing carcasses than conformation system in both FT and TT carcasses.

### 4.4 EUROP Conformation Class and Meat Quality Characteristics

In present study, it was found that as the CC increased, no significant difference occurred between the meat quality parameters of three different muscles in FT lambs. Similar findings were obtained in TT lambs, and in addition, it was determined that expressed juice tended to increase as CC increased from 8 to 9. Supporting these results, Wajda and Daszkiewicz (2000) found that there were no significant differences among the bull conformation classes such as U, R and O, either in physiochemical characteristics (pH, colour brightness, water‐holding capacity) or in sensory characteristics. Janiszewski et al. (2018) found that there were no differences between the CCs in terms of pH_24_ and the meat lightness and other meat colour parameters in young bull carcasses. In the same study, it was reported that meat redness and yellowness colour were lower in meat from P grade carcasses than in other grades. In studies conducted on TT lambs (Cañeque et al. 2001; Santos‐Silva et al. 2002; Díaz et al. 2003; Ekiz et al. 2019), it was reported that as slaughter weight and CC increased, there was a decrease in the L* value and an increase in the a* value. The findings of the present study were not consistent with the reported values ​​for L* and a*. In addition, Ekiz et al. (2024) reported that the CC was not significant on most meat quality traits of Akkaraman lambs. The findings of the present study are compatible with the findings of Ekiz et al. (2024).

The adult ewe live weight is 45–50, 40–45 and 35–40 kg in Akkaraman, Herik and Karayaka breeds, respectively (Akcapınar 2000; TAGEM 2009). In the present study, there were three conformation and fatness classes in the FT sheep carcass group and two classes in the TT sheep carcass group. It is thought that the reason for the formation of different conformation and fatness classes in the FT carcass group despite similar pre‐slaughter and slaughter weights is due to the presence of two different breeds in this group. In spite of having a similar carcass weight, breed can affect conformation and fatness class (Sanudo et al. 1997; Schreurs and Kenyon 2017; Caro et al. 2018). There were two different classes in conformation and fatness groups in the TT carcass group. This result could be explained by individual differences among Karayaka lambs.

The European carcass classification system was originally developed for European TT sheep breeds. Since the distribution of fat tissue in TT lambs differs from in FT lamb, this may create a disadvantage regarding the effectiveness of the ECCS for FT breeds.

In the present study, it was found that the FC increased in FT lamb carcasses, total carcass fat percentage, subcutaneous fat percentage and intermuscular fat percentage increased, while total non‐carcass fat percentage, trimmed meat percentage, bone percentage, muscle/total fat ratio and muscle/carcass fat ratio decreased.

When the European conformation system was compared in terms of distinguishing carcasses of FT and TT lambs slaughtered at the same live weight, it was determined that it was successful in terms of trimmed meat percentage in FT lamb carcasses but not in TT lamb carcasses. In terms of carcass characteristics, it was determined that the European fatness classification system was more successful in distinguishing carcasses than conformation system both FT and TT carcasses. In this study, it was noteworthy that similar to the findings in the FC, as the CC increased, the testicular fat percentage increased in both FT and TT lambs.

The effect of fatness on meat quality traits was significant only in a*, b* and expressed juice traits in TT lambs, while all meat quality parameters were insignificant in FT lambs. While the effect of conformation on carcass traits was significant in some meat composition traits in FT lambs, the effect of this class on meat quality traits was insignificant in both tail structures. In conclusion, in FT and TT lambs, the ECCS is especially effective in distinguishing carcass characteristics according to fatness, but this system could be improved for FT and TT lamb carcasses in terms of meat quality characteristics.

## Author Contributions

**Conceptualisation**: Bulent Teke. **Methodology**: Bulent Teke. **Investigation**: Bulent Teke, Akif Uysal, Mustafa Ugurlu, Buket Nacar, Deniz Ay and Filiz Akdag. **Validation**: Akif Uysal, Mustafa Ugurlu, Buket Nacar, Deniz Ay and Filiz Akdag**. Resources**: Akif Uysal, Mustafa Ugurlu, Buket Nacar, Deniz Ay and Filiz Akdag. **Writing–original draft**: Bulent Teke and Filiz Akdag. **Writing–review and editing**: Bulent Teke, Akif Uysal, Mustafa Ugurlu, Buket Nacar, Deniz Ay and Filiz Akdag.

## Ethics Statement

The study protocol of the present study was accepted by the Ethics Committee of Ondokuz Mayis University, Samsun, Turkey (Approval number: 2019/54).

## Conflicts of Interest

The authors declare that no conflicts of interest.

## Peer Review

The peer review history for this article is available at https://www.webofscience.com/api/gateway/wos/peer‐review/10.1002/vms3.70449.

## Data Availability

The data that support the findings of this study are available from the corresponding author upon reasonable request.
